# Transcriptome Analysis Revealed the Mechanism of Nitrate Absorption in Tea Plants

**DOI:** 10.3390/plants15091352

**Published:** 2026-04-28

**Authors:** Weiwei Deng, Qiangqiang Xiong, Kang Wei, Yongxin Wang, Liyuan Wang

**Affiliations:** 1 State Key Laboratory of Tea Plant Germplasm Innovation and Resource Utilization, Anhui Agricultural University, Hefei 230036, China; dengweiwei@ahau.edu.cn (W.D.); xqq@stu.ahau.edu.cn (Q.X.); 2 Breeding of Special Economic Animals and Plants, Ministry of Agriculture and Rural Affairs, Tea Research Institute, Chinese Academy of Agricultural Sciences, Hangzhou 310008, China; weikang@mail.tricaas.com

**Keywords:** *Camellia sinensis*, gene expression, nitrate uptake, transcriptome

## Abstract

Nitrate (NO_3_^−^) serves as a critical nitrogen source and signaling molecule essential for its growth and quality formation. Although substantial genetic variation in nitrogen use efficiency (NUE) has been documented among tea cultivars, a systematic characterization of nitrate (NO_3_^−^) absorption kinetics and the associated genome-wide transcriptional regulatory networks across varying nitrate concentrations remains lacking. This study employed ^15^N isotope labeling and transcriptome sequencing to systematically analyze the absorption characteristics and molecular response mechanisms of the cultivars ‘Longjing 43’ and ‘Zhongming 6 hao’ under varying NO_3_^−^ concentrations. Results revealed significant differentiation in absorption strategies: ‘Zhongming 6 hao’ exhibited a significantly higher absorption rate at low concentrations, whereas ‘Longjing 43’ demonstrated enhanced performance at high concentrations. Transcriptome analysis indicated that both cultivars shared coordinated regulation of ‘photosynthesis’ and ‘nitrogen metabolism’ pathways. Furthermore, 14 nitrogen metabolism genes and 64 differentially expressed transcription factors (including MYB, NAC, and LBD families) were identified. Specifically, the *CsNiR* gene (encoding nitrite reductase) was functionally validated; silencing of *CsNiR* significantly reduced nitrite reductase activity, confirming its positive regulatory role. This study provided a theoretical framework and key candidate genes for breeding nitrogen-use-efficient varieties, which is essential for sustainable tea production.

## 1. Introduction

The tea plant (*Camellia sinensis* (L.) O. Kuntze) is a vital perennial economic crop. Free amino acids (particularly theanine) and secondary metabolites such as catechins, which accumulate in tender shoots, constitute the core determinants of tea quality [1,2,3]. As a primary limiting nutrient, nitrogen (N) directly governs tea plant growth, development, and quality formation, exerting a profound influence on yield, flavor profile, and economic returns [2,4]. In tea garden management, nitrogen fertilizer application has long exceeded the actual uptake capacity of tea plants, leading to resource inefficiency and exacerbated environmental pollution [5]. Therefore, an in-depth understanding of the mechanisms underlying nitrogen absorption, assimilation, and regulation in tea plants holds substantial theoretical and practical significance for enabling precision fertilization, enhancing tea quality, and promoting sustainable production.

Extensive research has established that the tea plant is a prototypical ‘ammonium-preferring’ species [6,7]. When both NH_4_^+^ and NO_3_^−^ are available in the rhizosphere, tea roots exhibit preferential and more efficient uptake of NH_4_^+^ [8]. Numerous studies have consistently demonstrated that, under either single or mixed nitrogen source conditions, the root uptake rate of NH_4_^+^ significantly surpasses that of NO_3_^−^ [7,9]. Nevertheless, although NH_4_^+^ serves as the predominant preferred nitrogen source, NO_3_^−^, a widely distributed inorganic nitrogen form in soils, plays an indispensable role in nitrogen nutrition and quality-related metabolism in tea plants [7,10]. Moreover, NO_3_^−^ functions not only as a nutrient but also as a signaling molecule, modulating the expression of multiple genes involved in nitrogen metabolism and thereby influencing plant growth and development [11].

Nitrate (NO_3_^−^) uptake in plants is mediated by nitrate transporter proteins (NRTs). Based on their distinct substrate affinities, NRTs are mainly classified into two functional systems: the high-affinity transport system (HATS) and the low-affinity transport system (LATS) [12,13,14]. The NRT2 family proteins constitute the core components of HATS and play pivotal roles in NO_3_^−^ acquisition under low-concentration conditions. In contrast, LATS is primarily mediated by members of the nitrate transporter 1/peptide transporter (NPF, NRT1/PTR) family. Following the completion of the tea plant genome sequencing, multiple CsNRT family members have been identified. For instance, 109 NPF family genes have been annotated in the tea plant genome [15]. Transcriptome analyses further revealed that several *CsNRT* genes are transcriptionally induced in response to nitrogen signaling [16,17]. Functional validation via heterologous overexpression of *CsNRT2.4* in *Arabidopsis thaliana* demonstrated a significant enhancement in NO_3_^−^ uptake capacity in transgenic lines, providing direct experimental evidence supporting its role as a high-affinity nitrate transporter [17].

To date, systematic characterization of NO_3_^−^ absorption and associated transcriptional responses across varying nitrate concentrations remains lacking in tea plants. Moreover, although substantial genetic variation in nitrogen use efficiency (NUE) has been documented among tea cultivars, it remains unclear whether such variation arises from differences in NO_3_^−^ perception and gene regulatory responses [18,19,20]. In this study, we comparatively analyzed the NO_3_^−^ uptake rates of two tea cultivars ‘Longjing 43’ and ‘Zhongming 6 hao’, under differential nitrate treatments using ^15^NO_3_^−^ labeling assays. Concurrently, RNA-seq profiling was employed to dissect the molecular mechanisms underlying their transcriptional responses to varying NO_3_^−^ concentrations. This study initially elucidates the molecular basis of nitrate absorption in tea plants and offers candidate genes for developing high-nitrogen-efficiency varieties.

## 2. Results

### 2.1. Absorption of Different Concentrations of Nitrate by Tea Plants

**Figure 1:**
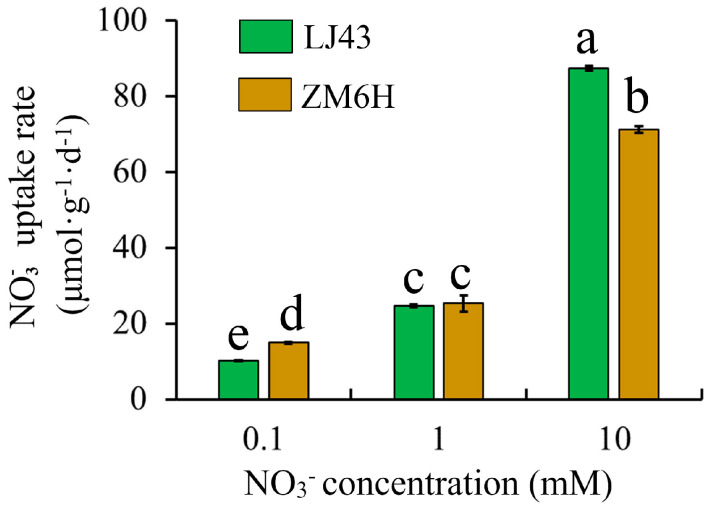
Nitrate uptake rates of tea cultivars ‘Longjing 43’ (LJ43) and ‘Zhongming 6 hao’ (ZM6H) under varying nitrate concentrations. Values represent three biological replicate means ± SD. Significant differences were detected using Duncan’s multiple-range test with SPSS 17.0 software. Different lowercase letters present significant differences (*p* < 0.05).

To investigate the uptake of nitrate at different concentrations by tea plants, ‘Longjing 43’ and ‘Zhongming 6 hao’ were treated with 0.1, 1, and 10 mM ^15^NO_3_^−^. As illustrated in Figure 1, the NO_3_^−^ absorption rate in both tea plant cultivars increased as the nitrate concentration increased. Under the 0.1 mM NO_3_^−^ treatment, the nitrate absorption rate of ‘Zhongming 6 hao’ was significantly higher than that of ‘Longjing 43’, reaching approximately 1.46 times that of the latter. At 1 mM NO_3_^−^, no significant difference in NO_3_^−^ uptake was observed between the two cultivars. However, under the 10 mM NO_3_^−^ treatment, ‘Longjing 43’ displayed a significantly higher nitrate absorption rate compared to ‘Zhongming 6 hao’.

### 2.2. Transcriptome Assembly and Identification of DEGs

In order to clarify the differences in the molecular mechanisms of nitrate absorption between the ‘Longjing 43’ and ‘Zhongming 6 hao’ tea cultivars, RNA-seq analysis was conducted on 24 samples (Table 1). The generated sequencing data exhibited high quality, with each sample yielding over 6 GB of data and Q30 scores ranging from 94.69% to 97.22%. These high-quality clean reads were subsequently aligned to the ~3.14 Gb tea plant reference genome, with mapping rates ranging from 68.52% to 77.76% across samples. In total, 61,817 genes were identified through this analysis.

**Figure 2:**
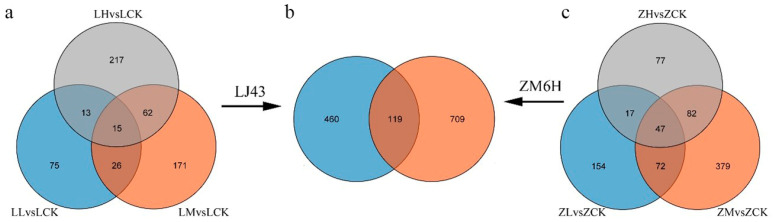
Identification of DEGs in the tea cultivars ‘Longjing 43’ and ‘Zhongming 6 hao’ in response to varying nitrate treatments. (**a**) ‘Longjing 43’; (**c**) ‘Zhongming 6 hao’; (**b**) intersection between ‘Longjing 43’ and ‘Zhongming 6 hao’.

Differentially expressed genes (DEGs) were identified based on DESeq2 criteria (|log_2_FoldChange| ≥ 1 and *p*adj < 0.05). In the nitrate treatment and control groups of tea plant ‘Longjing 43’, 129 (LLvsLCK), 274 (LMvsLCK), and 307 (LHvsLCK) DEGs were identified, respectively (Figure 2 and Table S1). In the nitrate treatment and control groups of ‘Zhongming 6 hao’, 290 (ZLvsZCK), 580 (ZMvsZCK), and 223 (ZHvsZCK) DEGs were identified, respectively. Among the DEGs, 119 were common to both ‘Longjing 43’ (total 579) and ‘Zhongming 6 hao’ (total 828). Notably, the number of DEGs in ‘Zhongming 6 hao’ under low nitrate concentration was higher than that in ‘Longjing 43’, which was consistent with the higher nitrate absorption rate of ‘Zhongming 6 hao’ under low nitrate concentration.

### 2.3. Gene Ontology and KEGG Enrichment Analysis of DEGs

To explore the response mechanism of varying nitrate concentrations, GO functional annotation and KEGG pathway enrichment analysis were conducted on 579 DEGs identified in ‘Longjing 43’ and 828 DEGs in ‘Zhongming 6 hao’.

**Figure 3:**
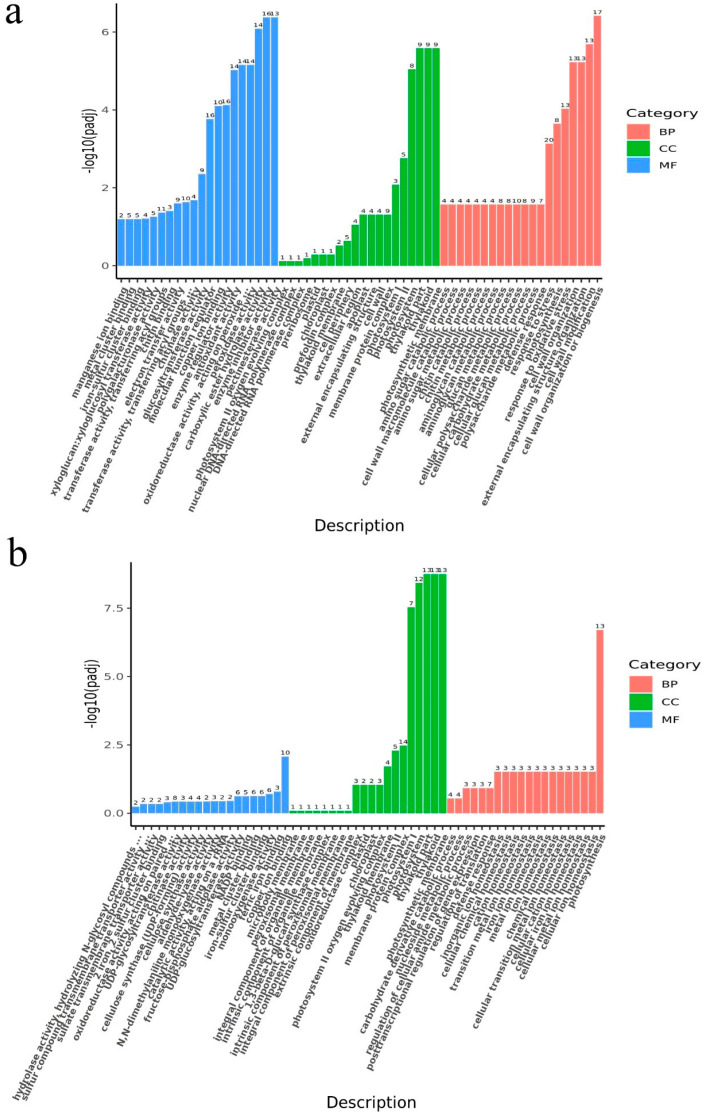
GO enrichment analysis of DEGs from ‘Longjing 43’ (**a**) and ‘Zhongming 6 hao’ (**b**) groups. Significantly enriched terms were defined by *p*adj < 0.05. The numbers above the bars indicate the number of DEGs enriched in each term.

As illustrated in Figure 3, the GO annotation results categorized the DEGs into three primary functional domains: Biological Process (BP), Molecular Function (MF), and Cellular Component (CC). In ‘Longjing 43’, the DEGs were predominantly enriched in the BP and MP categories. In contrast, the DEGs in ‘Zhongming 6 hao’ were more extensively distributed within the CC category.

**Figure 4:**
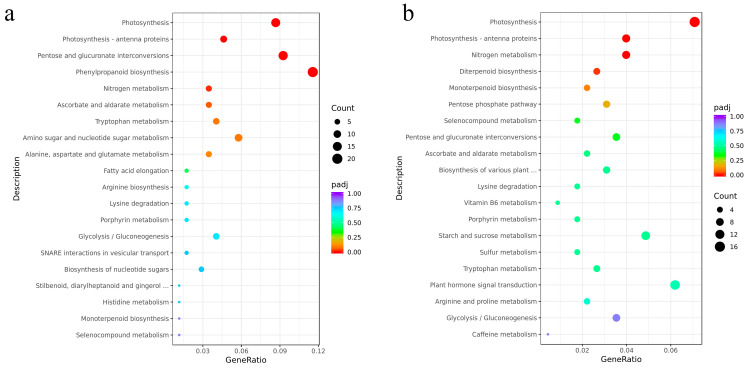
KEGG enrichment analysis of DEGs from ‘Longjing 43’ (**a**) and ‘Zhongming 6 hao’ (**b**) groups. Significantly enriched pathways were defined by *p*adj < 0.05.

KEGG pathway enrichment analysis further elucidated the metabolic pathway-level responses of the two tea plant cultivars (Figure 4). Both cultivars exhibited significant enrichment in key pathways including ‘Photosynthesis’, ‘Photosynthesis—antenna proteins’, and ‘Nitrogen metabolism’, suggesting that nitrate treatment influences photosynthetic efficiency and nitrogen assimilation capacity. However, each cultivar displayed distinct pathway enrichment patterns: ‘Longjing 43’ showed specific enrichment in ‘Pentose and glucuronate interconversions’, ‘Ascorbate and aldarate metabolism’, and ‘Phenylpropanoid biosynthesis’ pathway, which are closely linked to antioxidant defense, cell wall remodeling, and secondary metabolite production, whereas ‘Zhongming 6 hao’ was uniquely enriched in the ‘Diterpenoid biosynthesis’ pathway.

### 2.4. DEGs Involved in Nitrogen Absorption and Assimilation

**Figure 5:**
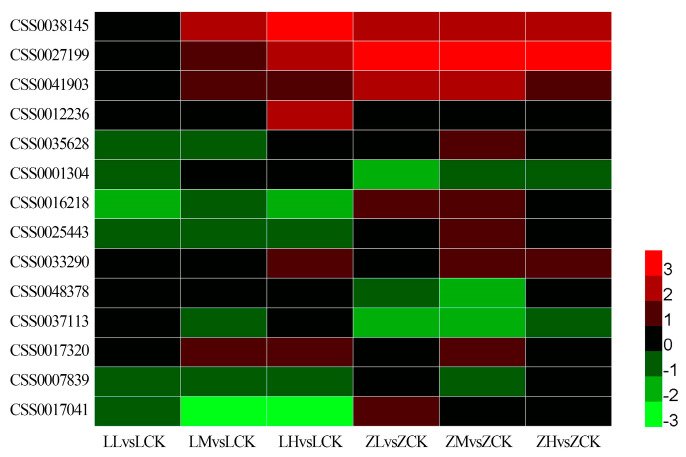
Expression analysis of DEGs involved in nitrogen absorption and assimilation. The different colors indicate DEG expression levels based on the log_2_FoldChange values. Red and green colors indicate upregulation and downregulation, respectively.

Among the 1288 identified DEGs, 14 genes were functionally annotated to nitrogen absorption and assimilation pathways (Figure 5 and Table S2). These included three genes encoding high-affinity nitrate transporters from the NRT2 family (CSS0041903, CSS0035628, CSS0001304), eight genes encoding low-affinity nitrate transporters from the NRT1 family, one gene encoding nitrate reductase (*NR*, CSS0027199), one gene encoding nitrite reductase (*NiR*, CSS0038145), and one gene encoding an ammonium transporter (AMT). Under low-nitrate treatment, none of those 14 genes were upregulated more than two-fold in ‘Longjing 43’. In contrast, five genes (CSS0038145, CSS0027199, CSS0041903, CSS0016218, and CSS0017041) in ‘Zhongming 6 hao’ exhibited an upregulation of more than two-fold. Under high-nitrate conditions, six genes (CSS0038145, CSS0027199, CSS0041903, CSS0012236, CSS0033290, and CSS0017320) were upregulated more than two-fold in Longjing 43, whereas only four genes (CSS0038145, CSS0027199, and CSS0041903, and CSS0033290) showed a similar level of upregulation in ‘Zhongming 6 hao’.

### 2.5. Analysis of Differentially Expressed Transcription Factors

**Figure 6:**
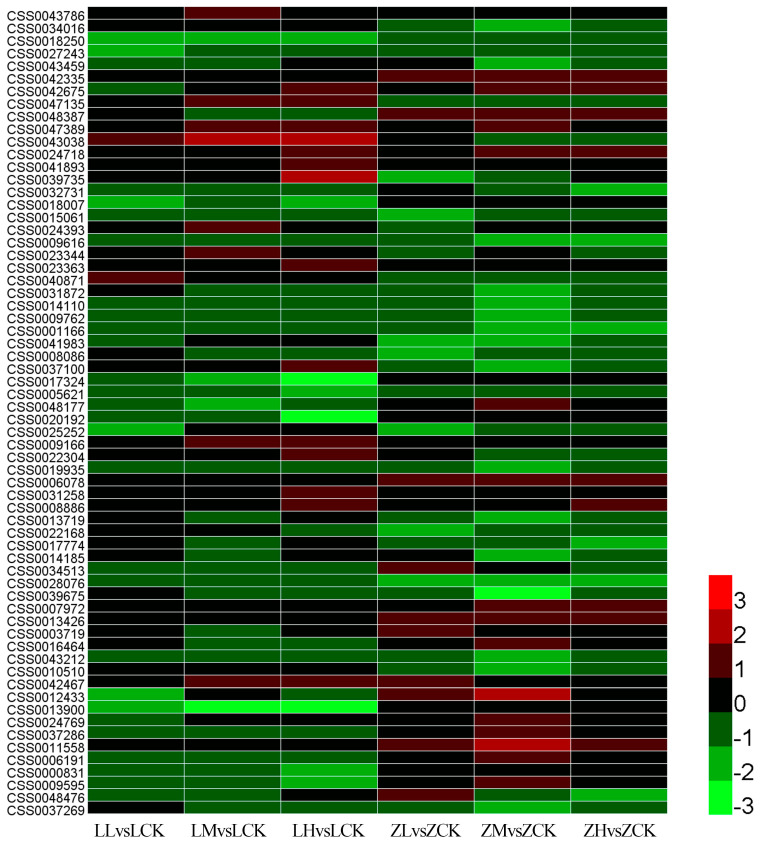
Expression analysis of differentially expressed transcription factors. The different colors indicate DEG expression levels based on the log_2_FoldChange values. Red and green colors indicate upregulation and downregulation, respectively.

Of the 1288 DEGs, 64 encoded transcription factors (Figure 6 and Table S3), accounting for approximately 4.97% of the total. These involved multiple families, specifically, five from the MYB family (CSS0043786, CSS0034016, CSS0018250, CSS0027243, CSS0043459), five from the NAC family (CSS0042335, CSS0042675, CSS0047135, CSS0048387, CSS0047389), four from the LBD (LATERAL ORGAN BOUNDARIES DOMAIN) family (CSS0043038, CSS0024718, CSS0041893, CSS0039735), and four from the Homeobox family (CSS0032731, CSS0018007, CSS0015061, CSS0024393). Additionally, DEGs encoding members of AP2/ERF, WRKY, GATA, and DOF families were also identified.

### 2.6. Validation of RNA-Seq Data by RT-qPCR

**Figure 7:**
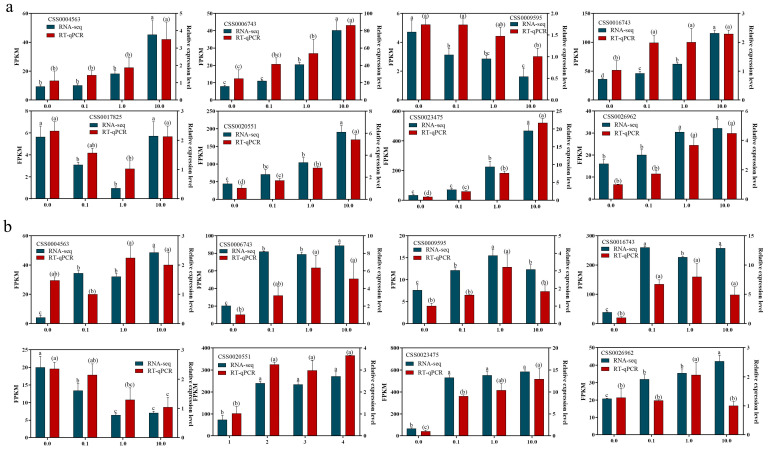
Validation of RNA-Seq results by RT-qPCR. (**a**) ‘Longjing 43’ groups. (**b**) ‘Zhongming 6 hao’ groups. Values represent three biological replicate means ± SD. The lowercase letters without parentheses present significant differences in RNA-seq data, and the lowercase letters in parentheses present significant differences in RT-qPCR data.

To validate the technical accuracy and reproducibility of the RNA-seq data, eight genes were randomly selected for RT-qPCR analysis (Figure 7). The results showed that the expression trends of these genes in RT-qPCR were highly consistent with the RNA-seq data, confirming the accuracy and credibility of the transcriptome profiling.

### 2.7. Validation of CsNiR Gene Function by Antisense Oligonucleotide Silencing

**Figure 8:**
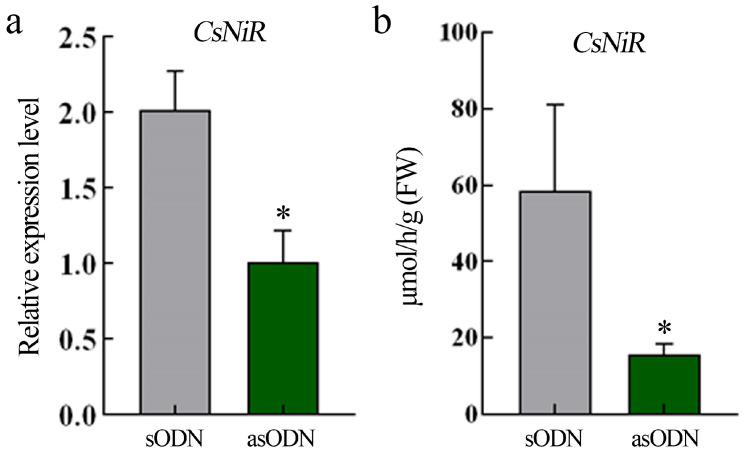
Antisense oligonucleotide silencing analysis of *CsNiR* in tea plants. (**a**) *CsNiR* expression level; (**b**) the nitrite reductase activity in RNAi treatment of *CsNiR*. Values represent three biological replicate means ± SD. * *p* < 0.05 (Student’s *t*-test).

To elucidate the function of the nitrite reductase gene (*NiR*, CSS0038145) in tea plants, an antisense oligodeoxynucleotide experiment was conducted. The results (Figure 8) showed that the expression level of the *CsNiR* gene treated with antisense oligonucleotides (AsODN) was significantly lower compared to those treated with sense oligonucleotides (sODN). Correspondingly, the nitrite reductase activity also decreased significantly.

## 3. Discussion

Nitrogen is an essential nutrient for plant growth and development [21,22]. Nitrogen deficiency induces profound physiological alterations, including inhibited growth, chlorosis, reduced yield, and decreased dry matter mass in crops [23,24,25,26]. As a leaf crop, tea plants have a high demand for nitrogen (N) fertilizers. However, excessive fertilizer application leads to resource waste and environmental pollution [5,27]. Understanding the mechanisms of nitrate absorption is therefore critical for improving nitrogen use efficiency.

**Figure 9:**
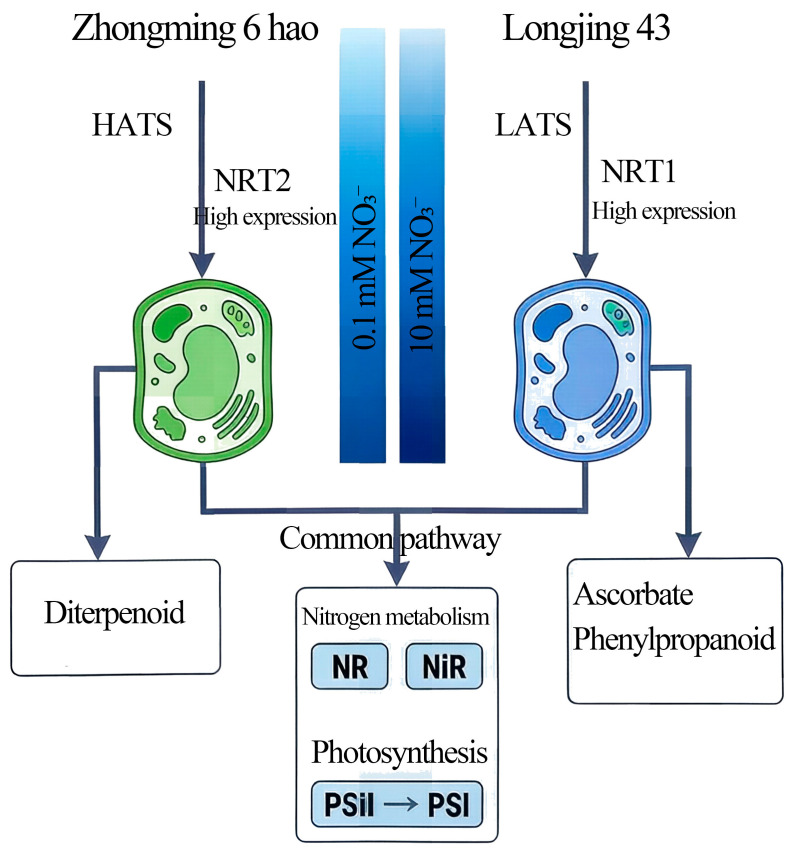
Schematic model of the regulatory mechanisms underlying differential nitrate absorption in tea plants. Under low-nitrate conditions, ‘Zhongming 6 hao’ showed higher nitrate uptake and a stronger transcriptional response, consistent with a greater contribution of high-affinity nitrate uptake-related processes. In contrast, under high-nitrate conditions, ‘Longjing 43’ showed higher nitrate uptake and stronger induction of genes associated with nitrate absorption and assimilation, consistent with a greater contribution of low-affinity nitrate uptake-related processes. Both cultivars shared enrichment in nitrogen metabolism and photosynthesis-related pathways, while cultivar-specific enrichment patterns suggested additional metabolic associations, such as diterpenoid biosynthesis in ‘Zhongming 6 hao’ and ascorbate- and phenylpropanoid-related metabolism in ‘Longjing 43’. These latter pathways may reflect cultivar-specific downstream metabolic adjustments rather than core nitrate uptake mechanisms and could represent additional traits of interest for future studies oriented toward improving nitrogen use efficiency in tea plants.

This study employed ^15^N isotope labeling and transcriptome sequencing to systematically analyze the absorption characteristics and molecular response mechanisms of ‘Longjing 43’ and ‘Zhongming 6 hao’ under varying nitrate concentrations. Results indicate that under low-concentration (0.1 mM) nitrate conditions, ‘Zhongming 6 hao’ exhibits a significantly higher absorption rate (approximately 1.46-fold) than ‘Longjing 43’. This suggests that ‘Zhongming 6 hao’ may possess a more efficient high-affinity nitrate transport system (HATS) (Figure 9). HATS is typically composed of members of the NRT2 family and the partner protein NAR2, and its activity can be rapidly induced by low concentrations of nitrate after nitrogen starvation [28,29]. This finding aligns with transcriptome data. Under 0.1 mM NO_3_^−^, ‘Zhongming 6 hao’ exhibited a higher number of DEGs. This transcriptional response correlates with its rapid sensing and higher absorption rate observed in the physiological experiments. Conversely, ‘Longjing 43’ exhibited superior absorption capacity under high nitrate concentrations (10 mM), approximately 1.23-fold higher than that of ‘Zhongming 6 hao’. Correspondingly, the number of DEGs identified in ‘Longjing 43’ was much higher than that in ‘Zhongming 6 hao’, indicating that its low-affinity transport system (LATS) may be more developed. LATS is mainly mediated by some members of the NRT1/PTR family, and these transporters exhibit high transport fluxes under high nitrate concentrations [30]. This complementary pattern of ‘Zhongming 6 hao’ being superior at low concentrations and ‘Longjing 43’ leading at high concentrations reveals the adaptive differentiation of the two tea plant varieties during evolution or artificial selection for different nitrogen environments. ‘Zhongming 6 hao’ may be more suitable for cultivation in soils with relatively poor nitrogen content, while ‘Longjing 43’ may have more advantages in intensive tea gardens with high nitrogen fertilizer input. The current study focused on short-term uptake kinetics (24 h) and early transcriptional responses (2 h), which are insufficient to observe biomass accumulation. The identified differences in absorption efficiency provide the physiological basis for long-term growth advantages.

While the two tea plant cultivars exhibit distinct nitrate uptake phenotypes, KEGG enrichment analysis indicates a potential convergence in the biological processes responding to nitrate. Both varieties showed significant enrichment in ‘Photosynthesis’, ‘Photosynthesis-antenna proteins’, and ‘Nitrogen metabolism’ pathways. This indicates that nitrate, acting as a signaling molecule, may play a crucial role in regulating the carbon–nitrogen balance. External nitrate treatment enhances photosynthetic capacity, providing sufficient reducing power and carbon skeletons for nitrogen assimilation [31,32]. This coordinated regulation ensures efficient conversion of nitrogen into organic compounds (e.g., amino acids) rather than toxic accumulation [10]. Distinct specificities were also observed. DEGs in ‘Longjing 43’ were significantly enriched in ‘Pentose and glucuronate interconversions’, ‘Ascorbate and aldarate metabolism’, and ‘Phenylpropanoid biosynthesis’, pathways related to antioxidant defense and secondary metabolite synthesis. This implies that ‘Longjing 43’ prioritizes defense and quality-related metabolism in response to nitrate. In contrast, ‘Zhongming 6 hao’ was uniquely enriched in ‘Diterpenoid biosynthesis’. Diterpenoids often serve as defense compounds or hormone precursors [33], suggesting that ‘Zhongming 6 hao’ activates defense or growth regulation mechanisms to survive in low-nitrogen environments. Furthermore, the GO analysis revealed distinct biological response strategies between the cultivars. In ‘Longjing 43’, DEGs were predominantly enriched in the BP and MP categories, suggesting that this cultivar responds to nitrate treatment by regulating metabolic activities and specific molecular functions, such as enzyme activity and binding. In contrast, the DEGs in ‘Zhongming 6 hao’ were more extensively distributed within the CC category, indicating that ‘Zhongming 6 hao’ may adapt to nitrate availability by modulating cellular architecture or subcellular localization. The metabolic differences may be caused by the long-term adaptation to different ecological environments or breeding goals of the two tea plant varieties.

Among the 1288 DEGs, 14 were annotated to nitrogen uptake and assimilation, including nitrate transport, reduction, and ammonium transport. Members of the NRT1 and NRT2 families exhibited distinct, concentration-dependent expression patterns, suggesting a finely tuned regulatory mechanism in tea roots. Consistent with previous findings where heterologous overexpression of *CsNRT2.4* enhanced root nitrate influx in Arabidopsis [17], our data highlight the importance of these transporters. While the number of DEGs differed between cultivars, the identification of specific high-affinity (*CsNRT2* family) and low-affinity (*CsNRT1* family) transporters, coupled with the distinct ^15^N uptake kinetics, suggests that transcriptional regulation of these transporters may be the primary determinant of the observed physiological efficiency differences in these cultivars. Nitrate reductase (NR) and nitrite reductase (NiR) serve as rate-limiting enzymes in the nitrogen assimilation cascade; consequently, their transcriptional regulation directly influences plant nitrogen use efficiency [34,35]. In this study, the differential expression of *NR* (CSS0027199) and *NiR* (CSS0038145) demonstrates that nitrate treatment modulates not only upstream nitrate acquisition but also downstream assimilatory metabolism. Antisense oligonucleotide silencing of *CsNiR* in tea plant shoots resulted in a significant reduction in nitrite reductase activity, confirming that *CsNiR* is functionally required for this enzymatic activity in plants. Although transcriptomic analysis focused on roots (nitrate uptake site), functional validation was conducted in shoots (primary assimilation site). Future work will prioritize validating this gene specifically in root tissues. Although tea plants are known to prefer ammonium ions, our data indicate that nitrate may serve as a crucial precursor for ammonium ion supply through the nitrate assimilation pathway. The identification of ammonium transporter (AMT) genes among the differentially expressed genes suggests a mechanism capable of rapidly taking up ammonium ions from both external sources and internal nitrate reduction.

Sixty-four differentially expressed transcription factor genes were identified, involving multiple families such as MYB, NAC, LBD, Homeobox, AP2/ERF, WRKY, GATA, and DOF. These transcription factors were identified as potential transcription factors involved in the nitrate response in tea plants. These candidates may play regulatory roles in modulating downstream nitrogen metabolism genes. MYB and NAC transcription factors are known to function extensively in plant nitrogen signal transduction and stress response [36,37,38]; the five members of each family identified here may mediate the tea plant nitrate response by regulating downstream nitrogen metabolism genes. Similarly, LBD family transcription factors are confirmed participants in nitrogen signaling and root development [39,40,41]; the differential expression of four LBD genes likely reflects morphological and physiological adaptations of tea roots to nitrate availability.

## 4. Materials and Methods

### 4.1. Plant Materials and Treatments

One-year-old vegetative cuttings of two tea cultivars, ‘Longjing 43’ and ‘Zhongming 6 hao’, served as experimental materials. ‘Longjing 43’ is a widely cultivated tea plant variety that thrives under conditions of sufficient nitrogen, while ‘Zhongming 6 hao’ is the latest selected tea tree variety that is resistant to low nitrogen. The plant materials were collected from the germplasm repository of the Tea Research Institute, Chinese Academy of Agricultural Sciences, Hangzhou, China. Seedlings were cultivated under natural light with temperatures maintained at 26/20 °C (day/night). The tea plants were grown in a complete nitrogen nutrient solution [42] for two months until the emergence of abundant adventitious roots. Subsequently, they were transferred to a nitrogen-free nutrient solution for a one-week nitrogen starvation treatment.

Tea plants were subjected to exogenous nitrate treatments at concentrations of 0, 0.1, 1, and 10 mM NO_3_^−^, with the nitrogen-starved group serving as the control (CK). Two hours were selected to investigate the early transcriptional responses induced by nitrate, as the expression of nitrate-responsive genes typically reaches a peak within 1 to 3 h after treatment. White new root samples were collected 2 h post-treatment for RNA extraction and transcriptome sequencing. RNA-seq analysis was repeated for three biological replicates. Samples were designated as LCK, LL, LM, and LH, corresponding to ‘Longjing 43’ treated with 0, 0.1, 1, and 10 mM NO_3_^−^, respectively. Similarly, ZCK, ZL, ZM, and ZH represented ‘Zhongming 6 hao’ under the respective treatments.

For uptake rate determination, tea plants were treated with 0.1, 1, and 10 mM ^15^NO_3_^−^ for 24 h, after which root samples were harvested for ^15^N isotope labeling analysis. Each sample was repeated for three biological replicates. Plant samples were oven-dried and homogenized into a fine powder. Total nitrogen concentration and δ^15^N isotope ratios were determined by a FlashEA 1112 Elemental Analyzer (Thermo Scientific, Waltham, MA, USA). The ^15^N atom% was derived from the δ^15^N values and was subsequently used to calculate the net nitrate uptake rate.

### 4.2. RNA Extraction and RNA Sequencing

Total RNA was extracted from root tissues using a plant RNA extraction kit (Aidlab Biotechnologies, Beijing, China). RNA concentration was assessed using a Qubit 2.0 fluorometer (Life Technologies, Carlsbad, CA, USA). RNA integrity and purity were evaluated using the Agilent 5400 Bioanalyzer (Agilent Technologies, Santa Clara, CA, USA), and the RNA Integrity Number (RIN) was above 7.5, indicating high integrity suitable for sequencing (Table S4). cDNA libraries were constructed using standard and strand-specific protocols. Library quality was evaluated using the Agilent 2100 Bioanalyzer system (Agilent Technologies, Santa Clara, CA, USA), followed by paired-end sequencing on the Illumina NovaSeq 6000 platform.

### 4.3. Read Mapping and Transcript Quantification

Raw sequencing data were quality-assessed using FastQC (v0.11.9). Adapter sequences, ambiguous nucleotides, and low-quality bases were trimmed using fastp to generate high-quality clean reads. Clean reads were aligned to the tea plant reference genome [43] (http://tpia.teaplant.org/) (accessed on 10 January 2025) using HISAT2 (v2.2.1). StringTie (v1.3.3b) was used for novel gene prediction. Based on alignment results, featureCounts (v2.0.6) quantified read counts for each gene, and FPKM values were calculated. Differential expression analysis was performed using DESeq2 R software (v1.16.1). Significantly differentially expressed genes (DEGs) were defined as those with |log_2_FoldChange| ≥ 1 and *p*adj < 0.05.

### 4.4. Differential Gene Enrichment Analysis

Gene Ontology (GO) functional enrichment analysis was conducted using the ClusterProfiler (v3.8.1) package, while pathway enrichment analysis utilized the KEGG database (https://www.kegg.jp/kegg/kegg1.html) (accessed on 12 June 2025). Enrichment results were visualized using the NovoMagic Cloud Platform (https://magic-plus.novogene.com/#/tool/list) (accessed on 12 June 2025), with *p*adj < 0.05 set as the threshold for significance.

### 4.5. RT-qPCR Validation

To validate RNA-seq accuracy, eight DEGs were randomly selected for RT-qPCR. Total RNA (1 μg) was used for first-strand cDNA synthesis with the PrimeScript™ RT Reagent Kit (TaKaRa, Dalian, China). The RT-qPCR reaction mixture (20 μL) contained 10 μL SYBR Premix Ex Taq™ (Takara, Japan), 2 μL diluted cDNA template, 0.4 μL each of forward and reverse primers, and 7.2 μL ddH_2_O. Reactions were performed on a 7500 Real-Time PCR System (Applied Biosystems, Waltham, MA, USA) under the following conditions: initial denaturation at 95 °C for 30 s, followed by 40 cycles of 95 °C for 10 s, 58 °C for 10 s, and 72 °C for 10 s. Primer sequences are listed in Table S5, with the tea plant *GAPDH* gene serving as the internal reference [44]. All samples were replicated three times independently, and relative gene expression levels were calculated using the 2^−ΔΔCt^ method.

### 4.6. Antisense Oligonucleotide Gene-Silencing Experiments

To validate the function of the differentially expressed *NIR* gene, antisense oligonucleotide (asODN) silencing was employed. Antisense oligonucleotide primers (Table S6) were designed using the Sfold platform (https://sfold.wadsworth.org/cgi-bin/soligo.pl) (accessed on 12 November 2025) and diluted to 20 μM. Young shoots (one bud and two leaves) of ‘Longjing 43’ were immersed in asODN and sODN solution and incubated for 48 h. After the treatment, the samples were quickly collected for the determination of gene expression levels and the enzymatic activity of NiR. Nitrite reductase (NiR) activity was assayed spectrophotometrically using the Grace Biotechnology (Suzhou) kit, following the manufacturer’s protocol. In brief, the reaction mixture containing enzyme extract and substrate was incubated under specific conditions, and the formation of nitrite was monitored by measuring the absorbance change at 540 nm. Enzyme activity was then calculated based on the standard curve.

## 5. Conclusions

This study elucidates the divergence in nitrate absorption strategies between ‘Longjing 43’ and ‘Zhongming 6 hao’: the former relies on the low-affinity system (LATS) to efficiently utilize high concentrations of NO_3_^−^, while the latter preferentially responds to low-nitrogen environments via the high-affinity system (HATS). Transcriptional analysis indicates that both varieties share common responses in nitrogen metabolism and photosynthesis pathways but exhibit specific regulation in secondary metabolism and defense-related pathways, suggesting the molecular basis for their long-term adaptation to different nitrogen environments. The differential expression of NRT family genes and transcription factors (such as MYB, NAC, LBD) collectively constitutes the regulatory network governing nitrate perception and utilization in tea plants. Silencing *CsNiR* expression in tea plants demonstrated that *CsNiR* positively regulates nitrite reductase activity.

## Figures and Tables

**Table 1:** Summary of RNA-seq quality and alignment statistics.

Sample	Total Raw Reads	Total Clean Reads	Q20 (%)	Q30 (%)	GC (%)	Total Mapped (%)
LCK1	48,785,472	47,947,276	97.99	94.84	44.31	76.19
LCK2	50,330,174	49,391,420	97.87	94.69	44.5	77.55
LCK3	49,948,310	49,008,218	97.97	94.75	44.73	77.71
LL1	49,598,776	48,658,570	98.06	95.09	45.11	69.92
LL2	46,865,688	46,030,610	98.06	95.06	44.97	73.2
LL3	46,088,752	45,191,424	97.89	94.72	44.89	68.52
LM1	48,552,602	47,639,844	97.95	94.87	44.71	74.16
LM2	49,814,436	48,819,950	98.09	95.15	44.1	74.06
LM3	48,082,842	47,471,600	98.88	96.78	44.43	75.21
LH1	46,931,364	46,073,982	98.07	95.12	44.74	71.08
LH2	47,471,922	46,396,630	97.93	94.71	44.85	73.37
LH3	54,685,204	53,045,182	99.11	97.22	44.64	77.76
ZCK1	45,967,178	44,968,128	97.97	94.87	44.73	73.17
ZCK2	44,604,126	43,619,408	97.94	94.74	45.62	71.01
ZCK3	42,267,274	41,197,538	98	94.92	45.39	72.07
ZL1	46,309,948	45,129,484	97.99	94.9	45.1	74.16
ZL2	41,406,716	40,595,786	98.09	94.98	45.1	73.79
ZL3	47,347,134	46,059,740	98.04	94.99	45.05	73.81
ZM1	46,194,160	44,955,516	97.97	94.86	45	76.31
ZM2	41,921,078	40,350,790	98.04	94.89	44.85	77
ZM3	49,164,604	46,679,666	98.02	94.94	44.74	76.61
ZH1	42,977,942	42,201,372	98.01	94.92	44.67	75.91
ZH2	42,336,854	41,477,278	98.03	94.95	45.16	77.03
ZH3	46,476,254	45,464,328	97.92	94.77	45.24	76.08

## Data Availability

The sequence data have been submitted to the NCBI Sequence Read Archive (https://www.ncbi.nlm.nih.gov/sra) (accessed on 10 April 2026) under BioProject accession number PRJNA1452536.

## Supplementary Materials

The following supporting information can be downloaded at https://www.mdpi.com/article/10.3390/plants15091352/s1, Table S1: Number of DEGs under varying nitrate concentrations. Table S2: Expression analysis of DEGs involved in nitrogen absorption and assimilation. Table S3: Expression analysis of DEGs involved in nitrogen absorption and assimilation. Table S4. The results of RNA quality. Table S5: Sequences of primers used for RT-qPCR. Table S6: Antisense oligonucleotide primers for the *CsNiR* gene.
